# Glutamate and GABA in Appetite Regulation

**DOI:** 10.3389/fendo.2013.00103

**Published:** 2013-08-15

**Authors:** Teresa C. Delgado

**Affiliations:** ^1^Intermediary Metabolism Group, Center for Neurosciences and Cell Biology of Coimbra, Coimbra, Portugal

**Keywords:** GABA, appetite, hypothalamus, NMR spectroscopy, glutamate

## Abstract

Appetite is regulated by a coordinated interplay between gut, adipose tissue, and brain. A primary site for the regulation of appetite is the hypothalamus where interaction between orexigenic neurons, expressing Neuropeptide Y/Agouti-related protein, and anorexigenic neurons, expressing Pro-opiomelanocortin cocaine/Amphetamine-related transcript, controls energy homeostasis. Within the hypothalamus, several peripheral signals have been shown to modulate the activity of these neurons, including the orexigenic peptide ghrelin and the anorexigenic hormones insulin and leptin. In addition to the accumulated knowledge on neuropeptide signaling, presence and function of amino acid neurotransmitters in key hypothalamic neurons brought a new light into appetite regulation. Therefore, the principal aim of this review will be to describe the current knowledge of the role of amino acid neurotransmitters in the mechanism of neuronal activation during appetite regulation and the associated neuronal-astrocytic metabolic coupling mechanisms. Glutamate and GABA dominate synaptic transmission in the hypothalamus and administration of their receptors agonists into hypothalamic nuclei stimulates feeding. By using ^13^C High-Resolution Magic Angle Spinning Nuclear Magnetic Resonance spectroscopy based analysis, the Cerdán group has shown that increased neuronal firing in mice hypothalamus, as triggered by appetite during the feeding-fasting paradigm, may stimulate the use of lactate as neuronal fuel leading to increased astrocytic glucose consumption and glycolysis. Moreover, fasted mice showed increased hypothalamic [2-^13^C]GABA content, which may be explained by the existence of GABAergic neurons in key appetite regulation hypothalamic nuclei. Interestingly, increased [2-^13^C]GABA concentration in the hypothalamus of fasted animals appears to result mainly from reduction in GABA metabolizing pathways, rather than increased GABA synthesis by augmented activity of the glutamate-glutamine-GABA cycle.

## Appetite Regulation: From the Periphery to the Hypothalamus

**Figure 1:**
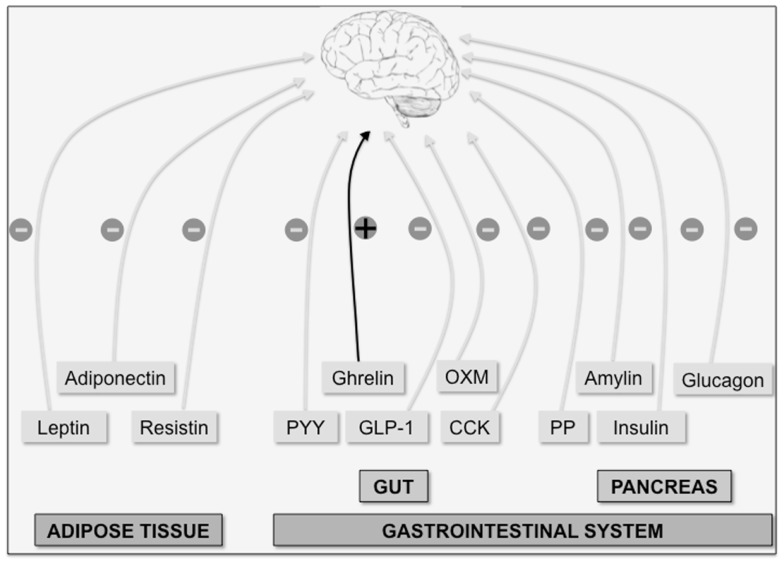
**The brain integrates multiple peripheral signals to control appetite**. Peripheral factors indicative of long-term energy whole-body status are produced by adipose tissue (leptin, adiponectin, and resistin). On the other hand, acute orexigenic (+) ghrelin signal (produced in the gut) and anorexigenic (−) signals such as the gut hormones peptide YY (PYY), oxyntomodulin (OXM), glucagon-like peptide-1 (GLP-1) and cholecystokinin (CCK), and the pancreatic hormones [insulin, glucagon, amylin, and pancreatic polypeptide (PP)] indicate long-term energy status.

Appetite is a highly regulated phenomenon, being hunger and satiety crucial factors in controlling food intake. Both food intake and energy expenditure disturbances lead to obesity, a pandemic syndrome frequently associated with the most prevalent and morbid pathologies in developed countries including heart disease, atherosclerosis, diabetes, and cancer (1). Appetite is closely regulated by a coordinated interplay between peripheral and central nervous system pathways. Two major groups of peripheral-derived signals inform the brain about the whole-body energy state: short-term signals produced by the gastrointestinal system and long-term signals produced by adipose tissue (Figure 1). There is a vast array of anorexigenic hormones causing loss of appetite secreted from the gut; these include: cholecystokinin (CCK) (2), glucagon-like peptide-1 (GLP-1) (3), peptide YY (PYY) (4), and oxyntomodulin (OXM) (5). Hormones derived from the pancreas, as pancreatic polypeptide (PP) (6), glucagon (7), insulin (8), and amylin (9), also exhibit anorexigenic actions. Finally, adipose tissue-derived anorexigenic signals, such as leptin (10), adiponectin (11), and resistin (12) have been described. On the other hand, gut-derived ghrelin is the only example of a peripheral hormone with orexigenic actions (13, 14), thereby increasing appetite upon its release usually before meals. In spite of intensive research during the last decades, other unidentified peripheral signals playing a role in appetite regulation probably exist. An increased knowledge on peripheral inputs controlling appetite could be relevant for the development of newly successful therapeutical approaches targeting obesity.

Studies employing either discrete lesions in the hypothalamus (15, 16) or surgical transection (17) of neural pathways have shown that central integration of peripheral-derived signals occurs mostly in the hypothalamus. The hypothalamus lies adjacent to three circumventricular organs, which are areas that permit substances to leave the brain without disrupting the blood-brain barrier (BBB), thereby permitting other substances that do not cross the BBB to exert their actions in the brain (18). In the last years, several neurotransmitters involved in hypothalamic appetite regulation have been identified [see, for example reviews (19–22)]. The cornerstone experiment for the identification of a potential neurotransmitter consists on the injection of the respective agent into the hypothalamus or adjacent ventricle of animal models and detection of a rapid increase or decrease in food intake. These experiments allowed not only the identification and characterization of several neurotransmitters involved in hypothalamic appetite regulation, but also to the precise tracking of pathways containing these signal molecules. Usually, neurotransmitters are classified into peptides, amino acids, and monoamines.

## Hypothalamic Peptidergic Neurotransmission and Appetite Regulation

In the arcuate nucleus of the hypothalamus, two sets of neuronal populations expressing either orexigenic neuropeptides [Neuropeptide Y (NPY) and Agouti-related peptide (AgRP)], or anorexigenic neuropeptides [Pro-opiomelanocortin (POMC) and Cocaine-amphetamine-related transcript (CART)] co-exist. Neuropeptide Y is synthesized in neurons situated in the far ventromedial aspect of the hypothalamic arcuate nucleus. Within the hypothalamus, NPY-expressing fibers project from the arcuate nucleus to the paraventricular nucleus, where the peptide is released (23). Thus, the administration of NPY to the hypothalamic paraventricular nucleus results in a robust and sustained increase of food intake in rodents (24), eventually leading to obesity when given repeatedly (25). On the other hand, antibody-mediated blockade of NPY action results in decreased food intake in starved animals (26). As neuronal populations expressing NPY are co-localized with AgRP-releasing neurons, the optogenetic (27) or pharmaco-genetic (28) stimulation of AgRP-expressing neurons also drives intense food intake whereas genetic ablation (29, 30) or pharmaco-genetic inhibition (28) decreases food consumption.

Neurons located mainly in the ventrolateral subdivision of the hypothalamic arcuate nucleus contain both the anorexigenic peptide CART and its precursor, POMC. The optogenetic stimulation of POMC-containing neurons reduces food intake (27) whereas genetic ablation of POMC-expressing cells (31, 32) increases appetite and food consumption. The gene encoding POMC gives rise to downstream peptide products, including melanocortins [adrenocorticotropic hormone (ACTH), the α-, β-, and γ-melanocyte-stimulating hormones (MSH) and β-endorphin (33). Release of the α-MSH peptide at the hypothalamic paraventricular nucleus potentially reduces food intake via activation of the melanocortin receptors, MC3R and MC4R. On the contrary, increased food intake and obesity are seen as a result of deletion of MC3R (34) and MC4R (35). In summary, activation of the POMC-expressing neurons in the hypothalamic arcuate nucleus triggers the release of α-MSH, which activates MC4R at the paraventricular nucleus, leading to suppressed food intake and increased energy expenditure. On the other hand, stimulation of AgRP-expressing neurons in the hypothalamic arcuate nucleus releases AgRP peptide, which antagonizes the effect of α-MSH on MC4R thereby increasing food intake.

To date, most effort has been placed on examining direct regulation of hypothalamic NPY/AgRP and POMC/CART-expressing neurons by various circulating factors whereas the role of upstream neural inputs has received comparatively less attention. This is surprising considering that both NPY/AgRP and POMC/CART neurons receive abundant excitatory and inhibitory synaptic input. The two neurotransmitters that account for most of the synaptic activity in the hypothalamus are the amino acids glutamate and γ-aminobutyric acid (GABA).

## Hypothalamic Glutamatergic Neurotransmission

### Neuronal-astrocytic glutamate metabolism

Glutamate is the dominant excitatory neurotransmitter in the central nervous system. In order for a neuron to release glutamate, the neurotransmitter must first be packed at high concentrations into synaptic vesicles, by means of specific vesicular glutamate transporters (VGLUT1, VGLUT2, and VGLUT3) (36). Upon stimulation, glutamate is released into the synaptic cleft to bind and elicit its effects on postsynaptic receptors, whether ionotropic [*N*-methyl-d-aspartate (NMDA), d,l-alpha-amino-3-hydroxy-5-methyl-isoxazole propionic acid (AMPA), kainic acid] or metabotropic receptors (mGluRs), present both in neurons and astrocytes.

Despite of its ubiquitous nature, extracellular glutamate levels are tightly regulated. The release of presynaptic glutamate largely exceeds the amount need for neurotransmission. As high glutamate concentrations could preclude further transmission or become associated with neurotoxicity events unless rapidly cleared, synaptically released glutamate is recycled from the extracellular space by means of excitatory amino acid transporters expressed predominantly on astrocytes (GLT-1 and GLAST). Within astrocytes, recycled glutamate can be metabolized to glutamine via glutamine synthetase or can be assimilated into the tricarboxylic acid (TCA) cycle. Glutamine released from astrocytes is further taken up again by neurons, where the mitochondrial phosphate-specific enzyme, glutaminase, reconverts inert glutamine-to-glutamate for subsequent repackaging into synaptic vesicles: the glutamate-glutamine cycle. Importantly, due to the lack of pyruvate carboxylase in neurons making them incapable of *de novo* synthesis of glutamate from glucose (37), the glutamate-glutamine cycle assures an adequate replenishment of glutamate in the central nervous system (38, 39). However, the glutamate-glutamine cycle faces a drain of compounds by oxidation (40–42), requiring a continuous replenishment of glutamate and glutamine in astrocytes. *De novo* synthesis of glutamate and glutamine by astrocytes requires an amino group, being that aspartate has been recently suggested as the neuron-born nitrogen donor (43). According to the astrocyte to neuron lactate shuttle hypothesis (ANLSH) (44, 45), energy requirements for astrocyte-mediated glutamate recycling are derived exclusively from the glycolytic glucose metabolism with the concomitant lactate production by astrocytes, the latter becoming the main oxidative fuel for neurons (44, 46, 47).

### Glutamate in appetite regulation

The intracerebroventricular injection (48), as well as the lateral hypothalamic injection of glutamate, or its excitatory amino acid agonists, kainic acid, AMPA, and NMDA (49), rapidly elicit an intense food intake in rats. Likewise, intracerebroventricularly injected mGluR5 agonists stimulate feeding in rodents whereas the mGluR5 receptor antagonist (R,S)-2-chloro-5-hydroxyphenylglycine, inhibits food intake (50). Although the above-mentioned studies implicate glutamate signaling in eliciting a stimulation of food intake, until recently the morphological examination of the glutamatergic system was difficult due to the lack of marker molecules specific to glutamatergic neurons. Two highly homologous transmembrane proteins, VGLUT1 and VGLUT2, have been proven to be specific for glutamatergic neurons. On this basis, several studies have identified the presence of a dense plexus of glutamatergic fibers in key hypothalamic areas involved in appetite regulation. For example, elevated expression of mRNA encoding VGLUT2 was found in neurons located in the ventromedial hypothalamus and from the ventrolateral aspect of the arcuate nucleus (51, 52). On the other hand, expression of VGLUT1 is confined to relatively weak labeling in the lateral hypothalamic area (51). Furthermore, by using double-labeling immunohistochemistry, the presence of VGLUT2 immunoreactivity has been shown in appetite-regulating POMC/CART-expressing neurons located in the arcuate nucleus (53, 54), where they receive glutamatergic input from neurons in the ventromedial nucleus of the hypothalamus (55). In addition, Kiss et al. provided evidences for the existence of glutamatergic innervation of NPY-expressing neurons in the rat hypothalamic arcuate nucleus (54).

To evaluate the role of glutamatergic input to NPY/AgRP and POMC/CART-expressing neurons, and more specifically its plasticity as regulated by glutamate NMDA receptors, Liu et al. generated mice lacking NMDA receptors on either AgRP or POMC neurons (56). The authors found that NMDA receptors on AgRP neurons, but not on POMC-expressing neurons, play a critical role in controlling energy balance indicating that fasting-induced activation of AgRP-releasing neurons is associated with markedly increased glutamatergic input (56). Furthermore, through the combination of cell-type-specific electrophysiological, pharmacological, and optogenetic techniques, Yang et al. found that food deprivation elevates excitatory synaptic input. According to these authors, gut-derived ghrelin acts at presynaptic receptors to increase glutamate release and activate NPY/AgRP-expressing neurons through ionotropic glutamate receptors (57).

In the last decade, astrocytes were reported to participate in several neuroendocrine processes although only recently their importance in the control of appetite and energy homeostasis has been established. Astrocytes express receptors for numerous neuropeptides, neurotransmitters, and growth factors, produce neuroactive substances, and express key enzymes necessary for sensing and processing nutritional signals. For example, the anorexigenic hormone leptin is known to affect astrocyte morphology and synaptic protein levels in the hypothalamus (58). Thereby, the observed diet-induced increase in leptin receptor levels in hypothalamic astrocytes is proposed to participate in the onset of obesity. More recently, Fuente-Martín et al. have shown that leptin directly modulates glutamate uptake in astrocytes in a time-dependent manner, stimulating a rapid increase that is downregulated with chronic exposure (59). The initial rapid increase in astrocyte’s glutamate captation indicates that leptin could reduce the stimulatory effects of glutamate at nearby synapses, thereby reducing appetite. In addition, when excess glutamate is released to the synaptic cleft, it is eventually recaptured by surrounding astrocytes, together with sodium ions, through the astrocytic glutamate cotransporter, GLAST. As a result, the intracellular sodium ions incorporated have to be extruded to the extracellular space, through the electrogenic Na^+^/K^+^ATPase and Na^+^K^+^2Cl^−^cotransporter, resulting in the intracellular incorporation of potassium ions. Increased intracellular potassium ions concentrations trigger an osmotically driven, aquaporin 4 (AQP4)-mediated, water transport culminating with astrocytic swelling (60). By using diffusion weighting imaging, Lizarbe et al. have recently shown significant increases in the slow diffusion parameters, consistent with astrocyte swelling response, in the hypothalamus of fasted relative to satiated animals (61, 62). On these grounds, we may hypothesize that, whereas an initial leptin-driven glutamate uptake in astrocytes shows anorexigenic potential (by diverting glutamate from neurons and thereby reducing glutamatergic neurotransmission), an excessive glutamate uptake by astrocytes, as occurs under orexigenic fasting conditions, causes astrocyte’s swelling and eventual response by amino release to the synaptic cleft (63) (augmenting glutamatergic neurotransmission associated with appetite enhancement).

## Hypothalamic GABAergic Neurotransmission

### Neuronal-astrocytic GABAergic metabolism

γ-Aminobutyric acid (GABA) is the main inhibitory neurotransmitter in the central nervous system. The regulation of GABA itself is achieved by several specialized molecular mechanisms mediating transport, sequestration, synthesis, and GABA degradation. GABAergic neurons express both mature isoforms of glutamate decarboxylase, GAD65 and GAD67, to convert the excitatory amino acid glutamate into GABA (64). Moreover, glutamine can be used as an alternative source of GABA. As described in the earlier section, the amino acid glutamine has long been known as the immediate precursor for glutamate. There is increasing evidence for a similar role of this glutamate-glutamine cycle in GABA synthesis [see review (65)]. GABA clearance from the synaptic cleft is mediated by specific, high-affinity, sodium- and chloride-dependent transporters, GAT1, GAT2, and GAT3 and the vesicular GABA transporter (VGAT) (66). After release, GABA elicits a biphasic response via activation of two classes of membrane receptors; either ionotropic (GABA_A_) or metabotropic (GABA_B_) receptors. Finally, it is estimated that more than 90% of all GABA in the mammalian central nervous system is degraded by transamination of GABA and α-ketoglutarate to succinic semialdehyde and glutamate in the mitochondria of astrocytes and neurons (67).

### GABA in appetite regulation

A stimulatory role for GABA in the regulation of hypothalamic controlled feeding behavior has been evidenced in the last years. The intracerebroventricular administration of the GABA_A_ receptor agonist, muscimol, stimulates feeding in satiated pigs, a response blockable by the specific GABA_A_ receptor antagonist, bicuculline (68). Also, systemic and intracerebroventricular administration of the GABA_B_ receptor agonist, baclofen, causes an increase in food intake in satiated pigs (69). Moreover, increased food intake obtained after administration of baclofen can be abolished by pretreatment with the GABA_B_ receptor antagonist, phaclofen (69). In agreement, several evidences indicate that neurons in the hypothalamic arcuate nucleus express to a large extent the GABA transporter, VGAT (70, 71) as well as the GABA synthesizing enzymes, GAD65 and GAD67 (70). Using immunohistochemistry, GAD65/GAD67 and GABA immunoreactivities have been demonstrated in the majority of NPY/AgRP neurons located in the hypothalamic arcuate nucleus (70, 71). On the other hand, in spite of GAD65/GAD67 mRNA presence has been demonstrated in approximately one-third of POMC-expressing neurons (72), VGAT was not detected in hypothalamic POMC cell bodies (53) suggesting the absence of POMC GABA-releasing neurons.

To further understand the role of GABAergic neurons in appetite regulation, Tong et al. have shown that while both NPY and AgRP stimulate food intake when infused into the brain, the weight loss seen when AgRP-expressing cells are destroyed is recapitulated by targeted deletion of their ability to release GABA, rather than NPY or AgRP (73). Furthermore, the severe anorectic phenotype induced by the diphtheria-induced acute ablation of AgRP-expressing neurons in adult mice can be rescued with chronic infusion of a benzodiazepine, known to enhance GABA effect at the level of GABA_A_ receptor (74). These evidences indicate that the synaptic release of GABA by AgRP-expressing neurons in the hypothalamic arcuate nucleus is required for normal regulation of energy balance. Wu et al. further explored the role of the GABAergic outputs of AgRP-expressing neurons. These authors found that in adult mice lacking AgRP-expressing neurons, pharmacological stimulation of GABA_A_ receptors in the parabrachial nucleus, by means of local injection of bretazenil (a partial GABA_A_ receptor agonist) is sufficient to maintain feeding. Wu and colleagues further corroborate these findings by examining the effects of either infusing a GABA antagonist directly into the parabrachial nucleus or selectively ablating AgRP inputs to this area. Both experiments induced a progressive decrease in food intake in mice, indicating that GABAergic inputs from arcuate nucleus AgRP-expressing neurons to the parabrachial nucleus are required to maintain a critical level of appetite stimulus (75). These observations clearly represent a potential shift away from early explanations of energy metabolism regulation, where GABA was thought to facilitate the feeding effect of NPY at target sites in the paraventricular nucleus by blocking opposing POMC transmission (76–78).

## Glutamate and GABA Actions on Neuronal-Astrocytic Metabolic Coupling Mechanism Underlying Hypothalamic Appetite Regulation

To date, glutamate and GABA actions on neuronal-astrocytic metabolic coupling mechanism underlying hypothalamic appetite regulation have been largely unexplored mainly due to the absence of appropriate *in vivo* methodological approaches. Earlier, a variety of *in vivo* Magnetic Resonance Imaging (MRI) and Magnetic Resonance Spectroscopy (MRS) methods have been shown to provide comprehensive information on cerebral activation and the underlying metabolic coupling mechanisms operating between neurons and astrocytes. However, the relatively large voxel size used in the acquisition of *in vivo* ^13^C Magnetic Resonance spectra precludes its use for studying the relatively reduced appetite controlling hypothalamic area of small rodents. Alternatively, High-Resolution ^13^C Nuclear Magnetic Resonance (NMR) spectroscopy investigations of the cerebral metabolism of tracers such as [1-^13^C]glucose or [2-^13^C]acetate contributed comprehensive information on the operation of the neuronal and astrocyte TCA cycles and the transcellular exchanges of glutamate–glutamine or GABA between neurons and astrocytes of the whole brain [see for example (79–82)].

Nevertheless, the relatively large amounts of cerebral tissue needed to prepare brain extracts for high-resolution ^13^C NMR spectroscopy constitutes an important limitation. To overcome the above-mentioned limitations, High-Resolution Magic Angle Spinning (HR-MAS) NMR spectroscopy, a technique yielding high quality spectra from very small tissue biopsies (5–10 mg, a size comparable to the size of the mice brain hypothalamus) was suggested to improve the spatial resolution and to investigate directly hypothalamic metabolism. Whereas ^1^H HR-MAS NMR has been used for metabolic profiling of normal and diseased tissues (83), ^13^C HR-MAS NMR spectroscopy offers the additional advantage of providing information on the operation of the metabolic pathways.

Recently, Violante et al. used ^13^C HR-MAS NMR spectroscopy analysis of mice hypothalamic biopsies, after [1-^13^C]glucose injection, to better understand the mechanisms underlying neurotransmission events and the associated neuronal-astrocytic metabolic coupling mechanisms underlying hypothalamic appetite regulation (84). Following [1-^13^C]glucose injection, glycolytic and TCA cycle intermediates are labeled distinctively, providing information on the relative contribution of the corresponding metabolic pathways. Initially [1-^13^C]glucose is metabolized to [3-^13^C]pyruvate via glycolysis. Labeled pyruvate is then reduced to [3-^13^C]lactate by lactate dehydrogenase, or alternatively enters the TCA cycle, producing [4-^13^C]glutamate and [4-^13^C]glutamine. Moreover [4-^13^C]glutamate can be converted to [2-^13^C]GABA. On this basis, the authors have shown that appetite stimulation, during the feeding-fasting paradigm, increases significantly the ^13^C incorporation in lactate carbons (84). Augmented lactate labeling most probably indicates a relatively increased glycolytic activity. Therefore, increased neuronal firing in the hypothalamus triggered by fasting may stimulate the use of lactate as neuronal fuel leading to increased astrocytic glucose consumption and glycolysis. Moreover, fasted mice show increased hypothalamic [2-^13^C]GABA content (84) most probably attributable to the existence of GABAergic neurons in key appetite regulation hypothalamic nuclei (70). Increased [2-^13^C]GABA concentrations may be derived either from increased net synthesis or reduced net degradation. Potential increases in GABA synthesis involve an augmented activity of the glutamate-glutamine-GABA cycle, since glutamate and glutamine are considered the main precursors of GABA. Interestingly, despite the increase in the [2-^13^C]GABA enrichment no increase was detected in [4-^13^C]glutamate or [4-^13^C]glutamine. Thus the increased [2-^13^C]GABA concentration in the hypothalamus of fasted animals appears to result mainly from reduction in GABA metabolizing pathways, rather than increased GABA synthesis by augmented activity of the glutamate-glutamine-GABA cycle.

**Figure 2:**
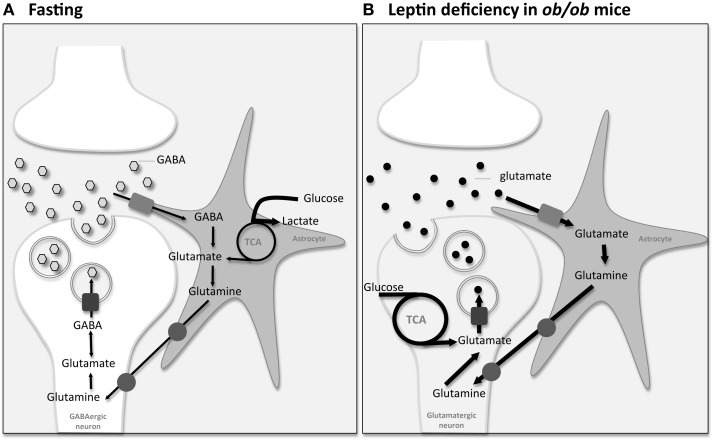
**Information on the integrated neuronal-astrocytic metabolic coupling mechanisms underlying appetite regulation can be investigated using [1-^13^C]glucose injection followed by analysis by ^13^C High-Resolution Magic Angle Spinning (HR-MAS) Nuclear Magnetic Resonance (NMR) spectroscopy analysis of mice hypothalamus biopsies**. **(A)** Fasting-induced changes: Violante et al. showed that increased neuronal firing in the hypothalamus triggered by fasting may stimulate the use of lactate as neuronal fuel leading to increased astrocytic glucose consumption and glycolysis (dark bold arrows). Moreover, fasted mice showed increased hypothalamic [2-^13^C]GABA content most probably attributable to the existence of GABAergic neurons in the hypothalamus. Despite elevated [2-^13^C]GABA, no increase was detected in the main precursors of GABA, glutamate, and glutamine, suggesting a reduction in GABA metabolizing pathways rather than increased GABA synthesis by augmented activity of the glutamate-glutamine-GABA cycle (84). **(B)** Leptin-deficiency-induced changes: we have shown that leptin deficiency in hyperphagic *ob/ob* mice resulted in significantly increased ^13^C incorporation from [1-^13^C]glucose in glutamate and glutamine carbons of hypothalamic biopsies suggesting that leptin-dependent appetite activation involves mainly increases on neuronal oxidation and glutamatergic neurotransmission together with elevated glutamate-glutamine cycling (dark bold arrows) (86).

Using a similar methodology, we have recently studied the neuronal-astrocytic metabolic coupling mechanism underlying hypothalamic appetite stimulation in hyperphagic leptin-deficient *ob/ob* mice. After a meal, leptin is released from adipose tissue to bind to the hypothalamic leptin receptor inducing an anorexigenic response consisting of a reduction in food intake and an increase in energy expenditure. On the contrary, in fasting periods, decreased plasma levels of leptin promote increased food intake and diminished energy consumption (85). Disruptions in the leptin signaling systems are often associated with hyperphagia and consequently obesity. In the leptin-deficient *ob/ob* mice, hypothalamic leptin signaling is drastically reduced and hyperphagia develops leading to obesity. We have showed that leptin deficiency in *ob/ob* mice resulted in significantly increased ^13^C incorporation from [1-^13^C]glucose in glutamate and glutamine carbons of hypothalamic biopsies suggesting that leptin-dependent hypothalamic activation, contrary to fasting-induced appetite stimulation, involves mainly increases in neuronal oxidation and glutamatergic neurotransmission together with elevated glutamate-glutamine cycling (86). Figure 2 provides an illustration on the use of ^13^C HR-MAS NMR spectroscopy to investigate appetite regulation in small hypothalamic areas during cerebral activation by different feeding activation paradigms. Unlike sensorial or motor paradigms [see for example (87–90)], where only glutamatergic or GABAergic terminals are involved in a simple activation/inhibition mechanisms, both glutamatergic and GABAergic stimulations on different neuronal populations may eventually lead to the dominant orexigenic or anorexigenic response, depending of their relative contributions. Most probably, the observed presence of both glutamatergic and GABAergic neurotransmission in association with different feeding activation paradigms may reflect the existence of complex feedback loops on the neuroendocrine regulation underlying appetite regulation. These feedback loops are crucial homeostatic mechanisms for the hypothalamic neuroendocrine regulation involving the operation of both peripheral signals and intrahypothalamic neurotransmitters.

## Concluding Remarks

Although to date most effort has been placed on examining direct regulation of hypothalamic appetite by neuropeptide-expressing neurons, it is evident that hypothalamic neurons further release and respond to excitatory and inhibitory amino acid neurotransmitters, as glutamate and GABA. Neuropeptides and amino acids neurotransmitters may both function as independent transmitters, or alternatively, neuropeptides may work by modulating the actions of glutamate and GABA and vice-versa. Herein, current knowledge on neuronal-astrocytic interactions underlying glutamate- and GABA-dependent hypothalamic appetite stimulation was reviewed. Apparently, different feeding paradigms associated with appetite stimulation account for different responses in neuronal-astrocytic metabolic coupling mechanisms. Whereas the fasting state is associated both with the use of lactate as neuronal fuel leading to increased astrocytic glucose consumption and with augmented hypothalamic GABAergic neurotransmission, elevated neuronal oxidation, and glutamatergic neurotransmission with increased glutamate-glutamine cycling are hallmarks of leptin deficiency in hyperphagic rodents. Further information on the neuronal-astrocytic metabolic pathways underlying appetite stimulation during different feeding paradigms, which can be achieved by the combined use of ^13^C HR-MAS NMR spectroscopy and metabolic tracers, may be a valuable tool for finding novel anti-obesity central-based therapeutical targets.

## Conflict of Interest Statement

The authors declare that the research was conducted in the absence of any commercial or financial relationships that could be construed as a potential conflict of interest.

## References

[1] Das UN Obesity: genes, brain, gut, and environment. Nutrition (2010) 26:459–73 10.1016/j.nut.2009.09.020 20022465

[2] Little TJ Horowitz M Feinle-Bisset C Role of cholecystokinin in appetite control and body weight regulation. Obes Rev (2005) 6:297–306 10.1111/j.1467-789X.2005.00212.x 16246215

[3] Gallwitz B Anorexigenic effects of GLP-1 and its analogues. Handb Exp Pharmacol (2012) 209:185–207 10.1007/978-3-642-24716-3_8 22249815

[4] Karra E Chandarana K Batterham RL The role of peptide YY in appetite regulation and obesity. J Physiol (2009) 587:19–25 10.1113/jphysiol.2008.164269 19064614 PMC2670018

[5] Dakin CL Small CJ Batterham RL Neary NM Cohen MA Patterson M Peripheral oxyntomodulin reduces food intake and body weight gain in rats. Endocrinology (2004) 145:2687–95 10.1210/en.2003-1338 15001546

[6] Hankir MK Parkinson JR Minnion JS Addison ML Bloom SR Bell JD Peptide YY 3-36 and pancreatic polypeptide differentially regulate hypothalamic neuronal activity in mice in vivo as measured by manganese-enhanced magnetic resonance imaging. J Neuroendocrinol (2011) 23:371–80 10.1111/j.1365-2826.2011.02111.x 21251093

[7] Geary N Pancreatic glucagon signals postprandial satiety. Neurosci Biobehav Rev (1990) 14:323–38 10.1016/S0149-7634(05)80042-9 2234610

[8] Werther GA Hogg A Oldfield BJ McKinley MJ Figdor R Allen AM Localization and characterization of insulin receptors in rat brain and pituitary gland using in vitro autoradiography and computerized densitometry. Endocrinology (1987) 121:1562–70 10.1210/endo-121-4-1562 3653038

[9] Cline MA Nandar W Smith ML Pittman BH Kelly M Rogers JO Amylin causes anorexigenic effects via the hypothalamus and brain stem in chicks. Regul Pept (2008) 146:140–6 10.1016/j.regpep.2007.09.003 17916389

[10] Schwartz MW Seeley RJ Campfield LA Burn P Baskin DG Identification of targets of leptin action in rat hypothalamus. J Clin Invest (1996) 98:1101–6 10.1172/JCI118891 8787671 PMC507530

[11] Qi Y Takahashi N Hileman SM Patel HR Berg AH Pajvani UB Adiponectin acts in the brain to decrease body weight. Nat Med (2004) 10:524–9 10.1038/nm0604-649a 15077108

[12] Tovar S Nogueiras R Tung LY Castaneda TR Vazquez MJ Morris A Central administration of resistin promotes short-term satiety in rats. Eur J Endocrinol (2005) 153:R1–5 10.1530/eje.1.01999 16131594

[13] Wren AM Small CJ Abbott CR Dhillo WS Seal LJ Cohen MA Ghrelin causes hyperphagia and obesity in rats. Diabetes (2001) 50:2540–7 10.2337/diabetes.50.11.2540 11679432

[14] Wren AM Seal LJ Cohen MA Brynes AE Frost GS Murphy KG Ghrelin enhances appetite and increases food intake in humans. J Clin Endocrinol Metab (2001) 86:5992 10.1210/jc.86.12.5992 11739476

[15] Hetherington HR Ranson SW Hypothalamic lesions and adiposity in the rat. Anat Rec (1940) 78:149–72 10.1002/ar.1090780203

[16] Leibowitz SF Hammer NJ Chang K Hypothalamic paraventricular nucleus lesions produce overeating and obesity in the rat. Physiol Behav (1981) 27:1031–40 10.1016/0031-9384(81)90366-8 7335803

[17] Grossman SP Grossman L Food and water intake in rats after transections of fibers en passage in the tegmentum. Physiol Behav (1977) 18:647–58 10.1016/0031-9384(77)90063-4 896975

[18] Ganong WF Circumventricular organs: definition and role in the regulation of endocrine and autonomic function. Clin Exp Pharmacol Physiol (2000) 27:422–7 10.1046/j.1440-1681.2000.03259.x 10831247

[19] Saper CB Chou TC Elmquist JK The need to feed: homeostatic and hedonic control of eating. Neuron (2002) 36:199–211 10.1016/S0896-6273(02)00969-8 12383777

[20] van den Pol AN Weighing the role of hypothalamic feeding neurotransmitters. Neuron (2003) 40:1059–61 10.1016/S0896-6273(03)00809-2 14687541

[21] Schwartz MW Woods SC Porte D Jr Seeley RJ Baskin DG Central nervous system control of food intake. Nature (2000) 404:661–71 10766253 10.1038/35007534

[22] Meister B Neurotransmitters in key neurons of the hypothalamus that regulate feeding behavior and body weight. Physiol Behav (2007) 92:263–71 10.1016/j.physbeh.2007.05.021 17586536

[23] Sawchenko PE Pfeiffer SW Ultrastructural localization of neuropeptide Y and galanin immunoreactivity in the paraventricular nucleus of the hypothalamus in the rat. Brain Res (1988) 474:231–45 10.1016/0006-8993(88)90438-6 2463058

[24] Stanley BG Kyrkouli SE Lampert S Leibowitz SF Neuropeptide Y chronically injected into the hypothalamus: a powerful neurochemical inducer of hyperphagia and obesity. Peptides (1986) 7:1189–92 10.1016/0196-9781(86)90149-X 3470711

[25] Zarjevski N Cusin I Vettor R Rohner-Jeanrenaud F Jeanrenaud B Chronic intracerebroventricular neuropeptide-Y administration to normal rats mimics hormonal and metabolic changes of obesity. Endocrinology (1993) 133:1753–8 10.1210/en.133.4.1753 8404618

[26] Lambert PD Wilding JP al-Dokhayel AA Bohuon C Comoy E Gilbey SG A role for neuropeptide-Y, dynorphin, and noradrenaline in the central control of food intake after food deprivation. Endocrinology (1993) 133:29–32 10.1210/en.133.1.29 8100519

[27] Aponte Y Atasoy D Sternson SM AGRP neurons are sufficient to orchestrate feeding behavior rapidly and without training. Nat Neurosci (2011) 14:351–5 10.1038/nn.2739 21209617 PMC3049940

[28] Krashes MJ Koda S Ye C Rogan SC Adams AC Cusher DS Rapid, reversible activation of AgRP neurons drives feeding behavior in mice. J Clin Invest (2011) 121:1424–8 10.1172/JCI46229 21364278 PMC3069789

[29] Gropp E Shanabrough M Borok E Xu AW Janoschek R Buch T Agouti-related peptide-expressing neurons are mandatory for feeding. Nat Neurosci (2005) 8:1289–91 10.1038/nn1548 16158063

[30] Bewick GA Gardiner JV Dhillo WS Kent AS White NE Webster Z Post-embryonic ablation of AgRP neurons in mice leads to a lean, hypophagic phenotype. FASEB J (2005) 19:1680–2 16099943 10.1096/fj.04-3434fje

[31] Yaswen L Diehl N Brennan MB Hochgeschwender U Obesity in the mouse model of pro-opiomelanocortin deficiency responds to peripheral melanocortin. Nat Med (1999) 5:1066–70 10.1038/12506 10470087

[32] Smart JL Tolle V Otero-Corchon V Low MJ Central dysregulation of the hypothalamic-pituitary-adrenal axis in neuron-specific proopiomelanocortin-deficient mice. Endocrinology (2007) 148:647–59 10.1210/en.2006-0990 17095588

[33] Mountjoy KG Functions for pro-opiomelanocortin-derived peptides in obesity and diabetes. Biochem J (2010) 428:305–24 10.1042/BJ20091957 20504281

[34] Butler AA Kesterson RA Khong K Cullen MJ Pelleymounter MA Dekoning J A unique metabolic syndrome causes obesity in the melanocortin-3 receptor-deficient mouse. Endocrinology (2000) 141:3518–21 10.1210/en.141.9.3518 10965927

[35] Huszar D Lynch CA Fairchild-Huntress V Dunmore JH Fang Q Berkemeier LR Targeted disruption of the melanocortin-4 receptor results in obesity in mice. Cell (1997) 88:131–41 10.1016/S0092-8674(00)81865-6 9019399

[36] Fremeau RT Jr Kam K Qureshi T Johnson J Copenhagen DR Storm-Mathisen J Vesicular glutamate transporters 1 and 2 target to functionally distinct synaptic release sites. Science (2004) 304:1815–9 10.1126/science.1097468 15118123

[37] Shank RP Bennett GS Freytag SO Campbell GL Pyruvate carboxylase: an astrocyte-specific enzyme implicated in the replenishment of amino acid neurotransmitter pools. Brain Res (1985) 329:364–7 10.1016/0006-8993(85)90552-9 3884090

[38] Cerdan S Kunnecke B Seelig J Cerebral metabolism of [1,2-13C2]acetate as detected by in vivo and in vitro 13C NMR. J Biol Chem (1990) 265:12916–26 1973931

[39] Erecinska M Silver IA Metabolism and role of glutamate in mammalian brain. Prog Neurobiol (1990) 35:245–96 10.1016/0301-0082(90)90013-7 1980745

[40] Rothman DL Behar KL Hyder F Shulman RG In vivo NMR studies of the glutamate neurotransmitter flux and neuroenergetics: implications for brain function. Annu Rev Physiol (2003) 65:401–27 10.1146/annurev.physiol.65.092101.142131 12524459

[41] Hertz L Kala G Energy metabolism in brain cells: effects of elevated ammonia concentrations. Metab Brain Dis (2007) 22:199–218 10.1007/s11011-007-9068-z 17882538

[42] Sonnewald U Westergaard N Petersen SB Unsgard G Schousboe A Metabolism of [U-13C]glutamate in astrocytes studied by 13C NMR spectroscopy: incorporation of more label into lactate than into glutamine demonstrates the importance of the tricarboxylic acid cycle. J Neurochem (1993) 61:1179–82 10.1111/j.1471-4159.1993.tb03641.x 8103082

[43] Pardo B Rodrigues TB Contreras L Garzon M Llorente-Folch I Kobayashi K Brain glutamine synthesis requires neuronal-born aspartate as amino donor for glial glutamate formation. J Cereb Blood Flow Metab (2011) 31:90–101 10.1038/jcbfm.2010.146 20736955 PMC3049464

[44] Pellerin L Magistretti PJ Glutamate uptake into astrocytes stimulates aerobic glycolysis: a mechanism coupling neuronal activity to glucose utilization. Proc Natl Acad Sci U S A (1994) 91:10625–9 10.1073/pnas.91.22.10625 7938003 PMC45074

[45] Tsacopoulos M Magistretti PJ Metabolic coupling between glia and neurons. J Neurosci (1996) 16:877–85 8558256 10.1523/JNEUROSCI.16-03-00877.1996 PMC6578818

[46] Pellerin L Bouzier-Sore AK Aubert A Serres S Merle M Costalat R Activity-dependent regulation of energy metabolism by astrocytes: an update. Glia (2007) 55:1251–62 10.1002/glia.20528 17659524

[47] Pellerin L Magistretti PJ Sweet sixteen for ANLS. J Cereb Blood Flow Metab (2012) 32:1152–66 10.1038/jcbfm.2011.149 22027938 PMC3390819

[48] Stricker-Krongrad A Beck B Nicolas JP Burlet C Central effects of monosodium glutamate on feeding behavior in adult Long-Evans rats. Pharmacol Biochem Behav (1992) 43:881–6 10.1016/0091-3057(92)90421-B 1448482

[49] Stanley BG Ha LH Spears LC Dee MG 2nd Lateral hypothalamic injections of glutamate, kainic acid, D,L-alpha-amino-3-hydroxy-5-methyl-isoxazole propionic acid or N-methyl-D-aspartic acid rapidly elicit intense transient eating in rats. Brain Res (1993) 613:88–95 10.1016/0006-8993(93)90458-Y 7688643

[50] Ploj K Albery-Larsdotter S Arlbrandt S Kjaer MB Skantze PM Storlien LH The metabotropic glutamate mGluR5 receptor agonist CHPG stimulates food intake. Neuroreport (2010) 21:704–8 10.1097/WNR.0b013e32833b4fe7 20505551

[51] Ziegler DR Cullinan WE Herman JP Distribution of vesicular glutamate transporter mRNA in rat hypothalamus. J Comp Neurol (2002) 448:217–29 10.1002/cne.10257 12115705

[52] Collin M Backberg M Ovesjo ML Fisone G Edwards RH Fujiyama F Plasma membrane and vesicular glutamate transporter mRNAs/proteins in hypothalamic neurons that regulate body weight. Eur J Neurosci (2003) 18:1265–78 10.1046/j.1460-9568.2003.02840.x 12956725

[53] Jarvie BC Hentges ST Expression of GABAergic and glutamatergic phenotypic markers in hypothalamic proopiomelanocortin neurons. J Comp Neurol (2012) 520:3863–76 10.1002/cne.23127 22522889 PMC4114021

[54] Kiss J Csaba Z Csaki A Halasz B Glutamatergic innervation of neuropeptide Y and pro-opiomelanocortin-containing neurons in the hypothalamic arcuate nucleus of the rat. Eur J Neurosci (2005) 21:2111–9 10.1111/j.1460-9568.2005.04012.x 15869507

[55] Sternson SM Shepherd GM Friedman JM Topographic mapping of VMH – arcuate nucleus microcircuits and their reorganization by fasting. Nat Neurosci (2005) 8:1356–63 10.1038/nn1550 16172601

[56] Liu T Kong D Shah BP Ye C Koda S Saunders A Fasting activation of AgRP neurons requires NMDA receptors and involves spinogenesis and increased excitatory tone. Neuron (2012) 73:511–22 10.1016/j.neuron.2011.11.027 22325203 PMC3278709

[57] Yang Y Atasoy D Su HH Sternson SM Hunger states switch a flip-flop memory circuit via a synaptic AMPK-dependent positive feedback loop. Cell (2011) 146:992–1003 10.1016/j.cell.2011.07.039 21925320 PMC3209501

[58] Garcia-Caceres C Fuente-Martin E Burgos-Ramos E Granado M Frago LM Barrios V Differential acute and chronic effects of leptin on hypothalamic astrocyte morphology and synaptic protein levels. Endocrinology (2011) 152:1809–18 10.1210/en.2010-1252 21343257 PMC3860256

[59] Fuente-Martín E Garcia-Caceres C Granado M de Ceballos ML Sanchez-Garrido MA Sarman B Leptin regulates glutamate and glucose transporters in hypothalamic astrocytes. J Clin Invest (2012) 122:3900–13 10.1172/JCI64102 23064363 PMC3484452

[60] Bender AS Schousboe A Reichelt W Norenberg MD Ionic mechanisms in glutamate-induced astrocyte swelling: role of K+ influx. J Neurosci Res (1998) 52:307–21 10.1002/(SICI)1097-4547(19980501)52:3<307::AID-JNR7>3.0.CO;2-H 9590439

[61] Lizarbe B Benitez A Pelaez Brioso GA Sanchez-Montanes M Lopez-Larrubia P Ballesteros P Hypothalamic metabolic compartmentation during appetite regulation as revealed by magnetic resonance imaging and spectroscopy methods. Front Neuroenergetics (2013) 5:6 10.3389/fnene.2013.00006 23781199 PMC3680712

[62] Lizarbe B Benitez A Sanchez-Montanes M Lago-Fernandez LF Garcia-Martin ML Lopez-Larrubia P Imaging hypothalamic activity using diffusion weighted magnetic resonance imaging in the mouse and human brain. Neuroimage (2013) 64:448–57 10.1016/j.neuroimage.2012.09.033 23000787

[63] Kimelberg HK Increased release of excitatory amino acids by the actions of ATP and peroxynitrite on volume-regulated anion channels (VRACs) in astrocytes. Neurochem Int (2004) 45:511–9 10.1016/j.neuint.2003.11.002 15186917

[64] Buddhala C Hsu CC Wu JY A novel mechanism for GABA synthesis and packaging into synaptic vesicles. Neurochem Int (2009) 55:9–12 10.1016/j.neuint.2009.01.020 19428801

[65] Schousboe A Waagepetersen HS Glial modulation of GABAergic and glutamat ergic neurotransmission. Curr Top Med Chem (2006) 6:929–34 10.2174/156802606777323719 16787266

[66] McIntire SL Reimer RJ Schuske K Edwards RH Jorgensen EM Identification and characterization of the vesicular GABA transporter. Nature (1997) 389:870–6 10.1038/39908 9349821

[67] Roth FC Draguhn A GABA metabolism and transport: effects on synaptic efficacy. Neural Plast (2012) 2012:805830 10.1155/2012/805830 22530158 PMC3316990

[68] Baldwin BA Ebenezer IS De La Riva C Effects of intracerebroventricular injection of muscimol or GABA on operant feeding in pigs. Physiol Behav (1990) 48:417–21 10.1016/0031-9384(90)90337-4 2176292

[69] Ebenezer IS Baldwin BA Effect of intracerebroventricular administration of the GABAB-receptor agonist baclofen on operant feeding in satiated pigs. Br J Pharmacol (1990) 101:559–62 10.1111/j.1476-5381.1990.tb14120.x 1963798 PMC1917733

[70] Ovesjo ML Gamstedt M Collin M Meister B GABAergic nature of hypothalamic leptin target neurones in the ventromedial arcuate nucleus. J Neuroendocrinol (2001) 13:505–16 10.1046/j.1365-2826.2001.00662.x 11412337

[71] Horvath TL Bechmann I Naftolin F Kalra SP Leranth C Heterogeneity in the neuropeptide Y-containing neurons of the rat arcuate nucleus: GABAergic and non-GABAergic subpopulations. Brain Res (1997) 756:283–6 10.1016/S0006-8993(97)00184-4 9187344

[72] Hentges ST Nishiyama M Overstreet LS Stenzel-Poore M Williams JT Low MJ GABA release from proopiomelanocortin neurons. J Neurosci (2004) 24:1578–83 10.1523/JNEUROSCI.3952-03.2004 14973227 PMC6730447

[73] Tong Q Ye CP Jones JE Elmquist JK Lowell BB Synaptic release of GABA by AgRP neurons is required for normal regulation of energy balance. Nat Neurosci (2008) 11:998–1000 10.1038/nn.2167 19160495 PMC2662585

[74] Wu Q Palmiter RD GABAergic signaling by AgRP neurons prevents anorexia via a melanocortin-independent mechanism. Eur J Pharmacol (2011) 660:21–7 10.1016/j.ejphar.2010.10.110 21211531 PMC3108334

[75] Wu Q Boyle MP Palmiter RD Loss of GABAergic signaling by AgRP neurons to the parabrachial nucleus leads to starvation. Cell (2009) 137:1225–34 10.1016/j.cell.2009.04.022 19563755 PMC2729323

[76] Cowley MA Smart JL Rubinstein M Cerdan MG Diano S Horvath TL Leptin activates anorexigenic POMC neurons through a neural network in the arcuate nucleus. Nature (2001) 411:480–4 10.1038/35078085 11373681

[77] Rao TL Kokare DM Sarkar S Khisti RT Chopde CT Subhedar N GABAergic agents prevent alpha-melanocyte stimulating hormone induced anxiety and anorexia in rats. Pharmacol Biochem Behav (2003) 76:417–23 10.1016/j.pbb.2003.08.016 14643840

[78] Millington GW The role of proopiomelanocortin (POMC) neurones in feeding behaviour. Nutr Metab (Lond) (2007) 4:18 10.1186/1743-7075-4-18 17764572 PMC2018708

[79] Gruetter R In vivo 13C NMR studies of compartmentalized cerebral carbohydrate metabolism. Neurochem Int (2002) 41:143–54 10.1016/S0197-0186(02)00034-7 12020614

[80] Garcia-Martin ML Garcia-Espinosa MA Ballesteros P Bruix M Cerdan S Hydrogen turnover and subcellular compartmentation of hepatic [2-(13)C]glutamate and [3-(13)C]aspartate as detected by (13)C NMR. J Biol Chem (2002) 277:7799–807 10.1074/jbc.M107501200 11744718

[81] Garcia-Espinosa MA Rodrigues TB Sierra A Benito M Fonseca C Gray HL Cerebral glucose metabolism and the glutamine cycle as detected by in vivo and in vitro 13C NMR spectroscopy. Neurochem Int (2004) 45:297–303 10.1016/j.neuint.2003.08.014 15145545

[82] Rodrigues TB Granado N Ortiz O Cerdan S Moratalla R Metabolic interactions between glutamatergic and dopaminergic neurotransmitter systems are mediated through D(1) dopamine receptors. J Neurosci Res (2007) 85:3284–93 10.1002/jnr.21302 17455302

[83] Holmes E Tsang TM Tabrizi SJ The application of NMR-based metabonomics in neurological disorders. NeuroRx (2006) 3:358–72 10.1016/j.nurx.2006.05.004 16815219 PMC3593384

[84] Violante IR Anastasovska J Sanchez-Canon GJ Rodrigues TB Righi V Nieto-Charques L Cerebral activation by fasting induces lactate accumulation in the hypothalamus. Magn Res Med (2009) 62:279–83 10.1002/mrm.22010 19526502

[85] Coll AP Farooqi IS O’Rahilly S The hormonal control of food intake. Cell (2007) 129:251–62 10.1016/j.cell.2007.04.001 17448988 PMC2202913

[86] Delgado TC Violante IR Nieto-Charques L Cerdan S Neuroglial metabolic compartmentation underlying leptin deficiency in the obese ob/ob mice as detected by magnetic resonance imaging and spectroscopy methods. J Cereb Blood Flow Metab (2011) 31:2257–66 10.1038/jcbfm.2011.134 21971349 PMC3323190

[87] Nandhu MS Paul J Kuruvila KP Abraham PM Antony S Paulose CS Glutamate and NMDA receptors activation leads to cerebellar dysfunction and impaired motor coordination in unilateral 6-hydroxydopamine lesioned Parkinson’s rat: functional recovery with bone marrow cells, serotonin and GABA. Mol Cell Biochem (2011) 353:47–57 10.1007/s11010-011-0773-x 21384157

[88] Floyer-Lea A Wylezinska M Kincses T Matthews PM Rapid modulation of GABA concentration in human sensorimotor cortex during motor learning. J Neurophysiol (2006) 95:1639–44 10.1152/jn.00346.2005 16221751

[89] Cartmell J Schoepp DD Regulation of neurotransmitter release by metabotropic glutamate receptors. J Neurochem (2000) 75:889–907 10.1046/j.1471-4159.2000.0750889.x 10936169

[90] Farrant M Nusser Z Variations on an inhibitory theme: phasic and tonic activation of GABA(A) receptors. Nat Rev Neurosci (2005) 6:215–29 10.1038/nrn1625 15738957

